# The Contribution of Cirrhosis Progression in Liver Dysfunction After Stereotactic Body Radiation Therapy

**DOI:** 10.1016/j.ijrobp.2025.07.1428

**Published:** 2025-07-23

**Authors:** Alex K. Bryant, John D. Rice, Emily Morris, Neehar D. Parikh, Kyle Cuneo, Laura A. Dawson, Teodor Stanescu, Charles Mayo, Theodore S. Lawrence, Matthew Schipper

**Affiliations:** a Department of Radiation Oncology, Veterans Affairs Ann Arbor Healthcare System, Ann Arbor, Michigan;; b Department of Radiation Oncology, University of Michigan, Ann Arbor, Michigan;; c Department of Biostatistics, University of Michigan, Ann Arbor, Michigan;; d Division of Gastroenterology and Hepatology, Department of Internal Medicine, University of Michigan, Ann Arbor, Michigan;; e Radiation Medicine Program, Princess Margaret Cancer Centre, Toronto, Ontario, Canada;; f Department of Radiation Oncology, University of Toronto, Toronto, Ontario, Canada

## Abstract

**Purpose:**

Patients with cirrhosis and hepatocellular carcinoma (HCC) often have progressive liver dysfunction after stereotactic body radiation therapy (SBRT), but the relative contribution of direct radiation toxicity versus cirrhosis progression is unknown. Our goal was to estimate the proportion of post-SBRT deterioration in the albumin-bilirubin (ALBI) score that is due to cirrhosis progression versus radiation toxicity.

**Methods and Materials:**

We first developed mixed-effects models to predict longitudinal ALBI trajectories among 6789 patients with cirrhosis within the University of Michigan system who did not have HCC. This resulted in a model for the expected change in ALBI over time due solely to cirrhosis progression. The model was then applied to a multi-institutional data set of 260 patients with cirrhosis and HCC treated with SBRT, resulting in patient-level predictions for ALBI deterioration due to cirrhosis progression alone. This predicted post-SBRT ALBI trajectory due to cirrhosis progression was then compared with the observed trajectory for each patient, resulting in an estimate of the proportion of post-SBRT ALBI change attributable to cirrhosis progression versus radiation toxicity.

**Results:**

In the cirrhosis cohort used for longitudinal modeling, ALBI trajectories were nonlinear, with an average 0.25-point improvement in the first year, followed by a worsening of 0.08 points on average per year. In the HCC cohort, the median baseline ALBI was −2.19 (unitless), reflecting a median albumin of 3.50 g/dL and a total bilirubin of 1.20 mg/dL. After SBRT, the mean ALBI increased (worsened) to −1.86 at 12 months. The estimated proportion of post-SBRT ALBI worsening due to cirrhosis was 14.2% (95% CI, 9.5%−18.8%) at 6 months and 24.9% (95% CI, 15.0%−34.7%) at 12 months, with the remainder attributed to SBRT.

**Conclusions:**

A significant proportion of post-SBRT liver function decline is due to the natural progression of cirrhotic liver dysfunction, and this proportion increases with time. These findings should improve estimates of SBRT treatment toxicity.

## Introduction

Among patients with cirrhosis, hepatocellular carcinoma (HCC) develops at an annual rate of ~2% to 3%.^1^ Liver dys-function in patients with HCC represents a complex interplay of underlying liver disease, tumor burden, and toxicity from liver-directed cancer therapies. When assessing posttreatment liver function in patients with successfully treated tumors, it is often difficult to separate the toxic effects of therapy from the natural decline that would have occurred without therapy due to cirrhosis progression. Disentangling these competing effects on posttreatment liver function—natural progression of cirrhosis and therapy—is critical to accurately describing the toxicity of liver-directed therapies and for building risk prediction models to drive treatment intensification or de-escalation.

The Child−Pugh (CP) score is the most commonly used tool for the assessment of liver function and prognostication in cirrhosis.^2,3^ Despite its clinical ubiquity, the CP score has notable limitations including the subjective elements of severity of ascites and hepatic encephalopathy, as well as lacking firm statistical grounding in its development.^2,4^ Given these limitations, the albumin-bilirubin (ALBI) score was recently proposed as a more objective and discriminatory measure of liver function in patients with HCC and is increasingly used to risk-stratify patients undergoing therapy for HCC.^5–8^ ALBI is a weighted sum of serum albumin and total bilirubin with lower (more negative) scores indicating better liver function. ALBI has also demonstrated prognostic power among the broader population of patients with cirrhosis without HCC.^9–11^

Accurately assessing HCC treatment toxicity requires a statistical model describing the expected ALBI trajectory without treatment, as any additional liver function decline beyond the expected trajectory could be attributed to treatment. Since most patients with nonmetastatic HCC will undergo treatment, and those who do not are a selected cohort with advanced disease, poor liver function, and advanced age,^12^ ALBI trajectories among untreated patients may not represent a valid comparison to estimate the effect of treatment. As such, separating the effects of HCC treatment from progression of cirrhosis requires a model of the trajectory of ALBI score among patients with cirrhosis without HCC.

In this study, we developed a longitudinal model using large-scale electronic medical record data to describe ALBI trajectories among a large population of patients with cirrhosis without HCC. We then applied this model to an independent cohort of patients with HCC treated with stereotactic body radiation therapy (SBRT)^13^ and compared observed versus predicted post-SBRT ALBI trajectories to estimate the proportion of the total ALBI worsening caused by cirrhosis versus radiation.

## Methods and Materials

### Data description and patient cohorts

For longitudinal modeling of ALBI trajectories, we first identified all patients with an inpatient or outpatient diagnosis of cirrhosis between 2008 and 2019 (International Classification of Diseases-9 [ICD-9]: 571.2, 571.5; ICD-10: K70.30, K70.31, K71.7, K74.60, K74.69) without a prior history of liver cancer or liver transplant (henceforth “cirrhosis cohort”) from the University of Michigan electronic medical record system using the Michigan Radiation Oncology Analytics Resource.^14^ We defined the index date as the first date of a cirrhosis diagnosis code entry in the health system. From a baseline population of 9351 patients, we excluded those with missing data on race, sex (male or female), and age, patients younger than 18 years, patients with missing baseline ALBI score (defined as the closest ALBI measurement within ± 60 days of the index date), and patients with only 1 ALBI measurement after the index date (Fig. E1). This resulted in a final cohort of 6789 patients.

The combined HCC data set from the University of Michigan and the Princess Margaret Cancer Centre has been described previously (Table 1).^13^ Briefly, this data set includes patients with HCC without vascular invasion, treated with photon SBRT at the University of Michigan and the American Joint Committee on Cancer [AJCC]. Eligibility criteria included patients with a histological or radiological diagnosis of T1, T2, or T3a HCC (AJCC TNM 7th edition) who were planned for SBRT and received a biologically effective dose of ≥45 Gy_10_, which is equivalent to 30 Gy in 6 fractions, the lowest dose fractionation schedule used at Princess Margaret Cancer Centre as per a prior large phase 1/2 trial.^15^ This study was approved by the local institutional review board and was conducted in accordance with both the Declarations of Helsinki and Istanbul. Written informed consent was waived.

### ALBI measurements and other covariates

Inpatient and outpatient laboratory measurements for the cirrhotic patients were ascertained from University of Michigan electronic health record and included serum albumin (g/dL) and total bilirubin (mg/dL). Baseline laboratory measurements were defined as the laboratory measurement closest to the date of cirrhosis diagnosis within a ± 60-day window. ALBI score was calculated as ALBI = −0.085*albumin + 0.66 log_10_(bilirubin),^5^ using albumin and total bilirubin measurements from the same day. If multiple albumin or bilirubin measurements were taken on the same day, their values were averaged before calculation of ALBI. Note that lower (more negative) ALBI scores represent better liver function. Other variables of interest were extracted from University of Michigan electronic medical record data and included age at cirrhosis diagnosis, sex (male, female), race (African American, Asian, White, and other), and type of cirrhosis (alcohol-related, hepatitis C virus [HCV], or not otherwise specified [NOS]). Alcohol-related cirrhosis was defined as the presence of a diagnosis code on the index date (ICD-9: 571.2; ICD-10: K70.30, K70.31) consistent with alcohol-related cirrhosis. Cirrhosis due to HCV was defined as the presence of a diagnosis code for HCV within ± 1 year of the index date. Other cirrhosis cases were classified as NOS and were assumed to predominantly represent cirrhosis due to metabolic associated steatohepatitis.

### Statistical analysis

To develop a model for longitudinal ALBI change in the cirrhosis cohort, we included all longitudinal ALBI measurements after the date of cirrhosis diagnosis through March 2020. To capture the natural history of ALBI among patients with cirrhosis without HCC or liver transplant, patients were censored at the time of HCC diagnosis or liver transplant and ALBI measurements after these events did not contribute to the analysis. Patients with only one ALBI measurement (eg, received care at other institutions) were excluded as longitudinal modeling is not possible with a single measurement. The longitudinal ALBI measurements were modeled with a mixed-effects generalized linear model with random intercepts and slopes to account for within-subject correlation in ALBI scores over time. Time was modeled as a linear spline to account for the observed initial improvement followed by gradual decline in ALBI over time and we include race and sex as covariates. Time-by-covariate interactions were also included to allow for varying time-trends in ALBI among patient subgroups. Variables of interest were prespecified and no variable selection was performed. The knot for the linear spline (in years) was selected by minimizing the Akaike Information criterion of candidate models, examined over a grid of potential values.

We sought to decompose post-SBRT change in ALBI into 2 components: (1) due to predicted cirrhosis progression and (2) due to SBRT toxicity. We used ≤4 measurement times per patient at 3, 6, 9, and 12 months post-SBRT, which matches our standard clinical follow-up. We then applied the linear spline model fitted to the cirrhosis data set to the patients with HCC: for each patient, we calculated the expected slope of the increase in ALBI would be if they had cirrhosis alone, that is, in the absence of HCC and radiation therapy (RT). This calculation was made under the assumption that they were ≥1-year postcirrhosis diagnosis, such that the trajectory of ALBI was expected to increase approximately linearly. The value of the predicted slope for each patient was then multiplied by the time points of interest to obtain predicted changes since baseline at each time point due to cirrhosis alone. We also calculated the observed change in ALBI at each post-SBRT timepoint by subtracting the baseline value from each follow-up value.

To obtain estimates and CIs for the decomposition of mean change in ALBI accounting appropriately for missing data, a linear mixed-effects regression model was fit to a data set including both the observed and predicted ALBI values. Main effects for time (4 levels) and type of ALBI measurement (predicted vs observed) and their interaction were included as main effects. Random effects were included for time (4 levels) at the patient level. The decomposition was obtained by taking the difference between this model’s fitted value for the observed ALBI (observed change) and its fitted value for the predicted ALBI (change due only to cirrhosis). CIs were obtained using the delta method. Importantly, although we expect the majority of post-SBRT liver function decline to be related to these 2 factors (radiation and cirrhosis progression), it is also likely that other factors—distant intrahepatic progression, local failure, systemic therapy, and additional liver-directed therapies—play a role, potentially inflating our estimates of the contribution of radiation. To address this, we performed multiple sensitivity analyses which refit the models in patient subsets that excluded those with local or distant intrahepatic failure, systemic therapy, and additional liver-directed therapies after SBRT. To assess the influence of radiation dose to the liver (mean liver dose) on our estimates, we performed additional subset analyses on patients with mean liver doses below versus above the sample median (7.5 Gy; physical dose to the liver-GTV volume). All statistical analyses were performed with R v4.2.1 (R Core Team).

## Results

### Cirrhosis and HCC cohorts

The cirrhosis cohort included 6789 patients diagnosed with cirrhosis from 2008 to 2020 (Table 2). There was a predominance of male patients (57%). Eight-four percent of the cohort was White and 7.8% was African American. Cirrhosis etiology was alcohol-related in 27%, HCV in 14%, and NOS in 59%. The median baseline ALBI score was − 2.19 (IQR, −2.79 to −1.54), reflecting a median baseline albumin of 3.60 (IQR, 3.00–4.10) and total bilirubin of 1.1 (IQR, 0.6–2.5). The median follow-up time was 1.7 years, and there was a median of 9 ALBI measurements per patient during follow-up (IQR, 4–19). The cumulative event rates of transplant and HCC diagnosis in our cohort are shown in Figure E2.

The HCC cohort included 260 patients (Table 1). The most common dose/fractionation schemes included 36 Gy in 3 fractions (14%), 50 Gy in 5 fractions (13.5%), 30 Gy in 3 fractions (12%), and 33 Gy in 3 fractions (11.5%). The mean dose to the liver-gross tumor volume (GTV) was 13 Gy (IQR, 7.5–18). All patients were treated with photon RT. Compared with the cirrhosis cohort, patients with HCC were slightly older (mean age 65 vs 62.4; *P* < .001) and were more likely to be male (77.7% vs 57.1%; *P* < .001). ALBI score was similar between the 2 cohorts (mean −2.12 for cirrhosis vs −2.10 for HCC;*P* = .61).

### Estimated ALBI trajectories by patient factors

**Fig. 1.**
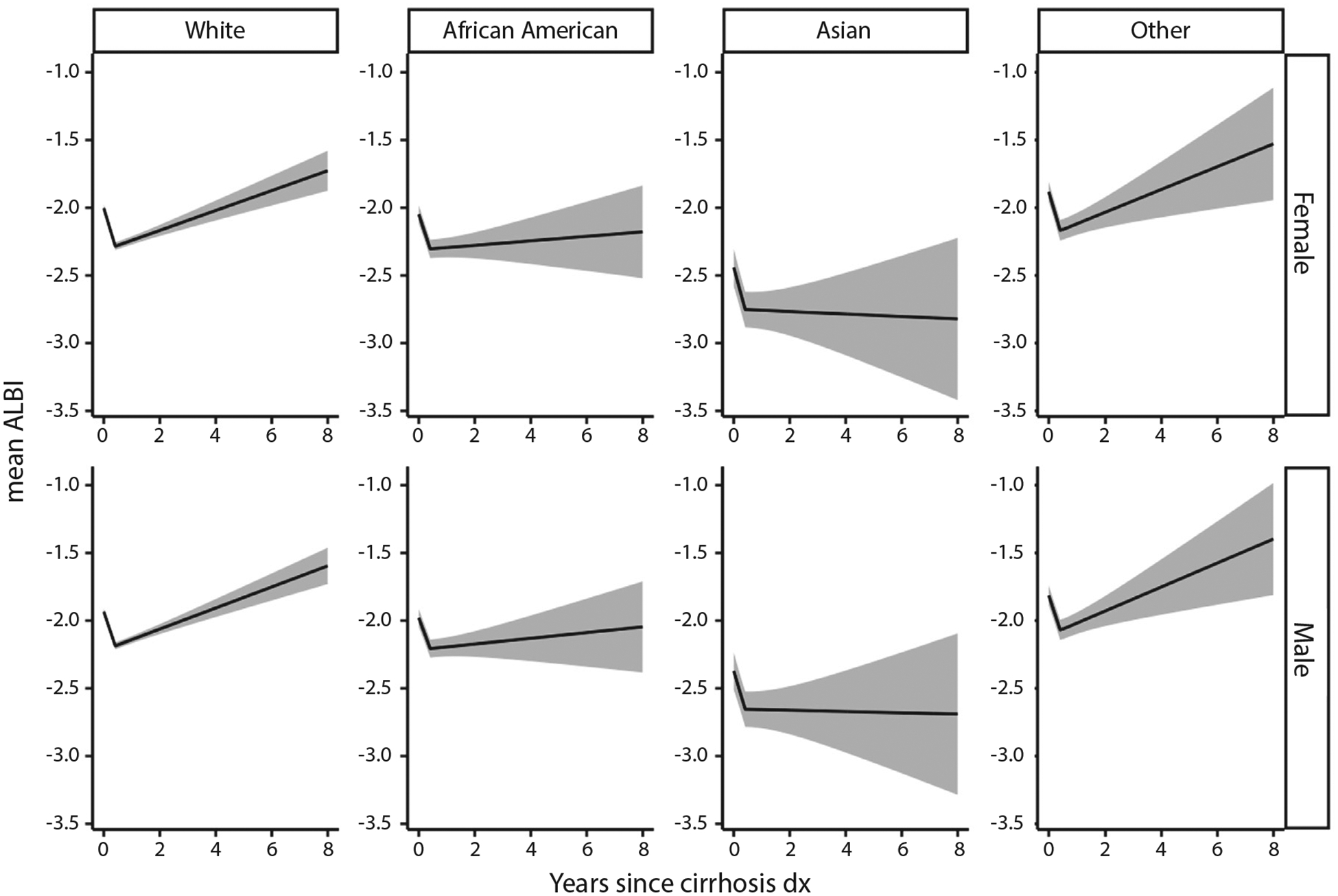
Estimated ALBI trajectories by sex and race among patients with cirrhosis and without HCC. The model included a linear split with a knot at 0.41 years (selected by the Akaike Information Criterion). Lower (more negative) ALBI scores reflect worse liver function. *Abbreviations:* ALBI = albumin-bilirubin score; dx = diagnosis; HCC = hepatocellular carcinoma.

Figure 1 shows model-estimated ALBI trajectories by race and sex subgroups for the cirrhosis cohort. On average, there was an estimated improvement of ~0.25 points in the first 5 months after cirrhosis diagnosis before slowly worsening at a rate of ~0.08 points per year. In the multivariable model, Asian race was associated with more favorable ALBI scores at diagnosis (–0.35 points; 95% CI, – 0.48 to – 0.22;*P* < .001) and a flatter trajectory (*P* for time interaction = .004) (Table E1). Male sex was associated with higher (worse) ALBI scores at diagnosis.

### Analysis of change in ALBI after SBRT

**Fig. 2.**
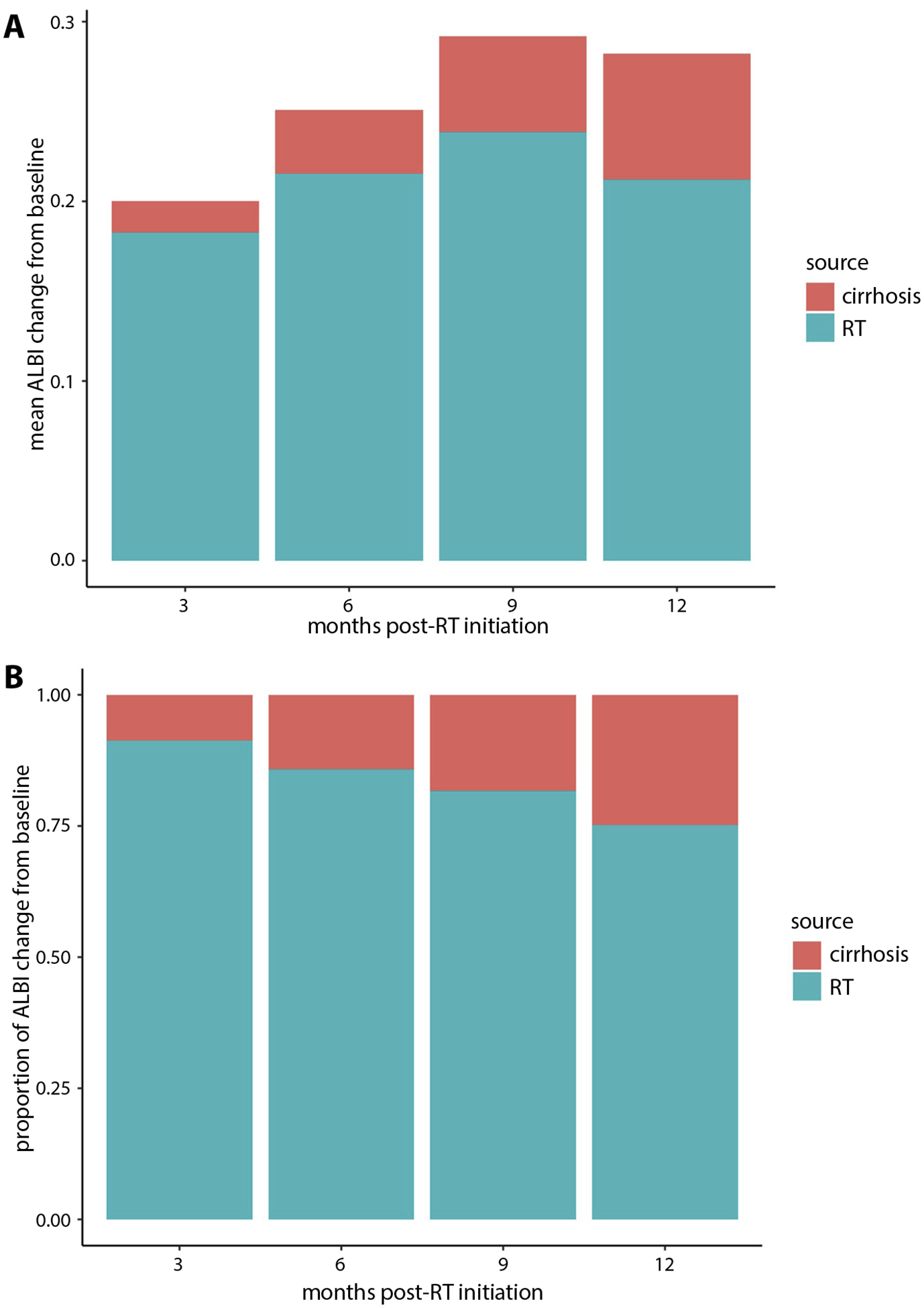
Decomposition of post-SBRT ALBI change. Predicted post-SBRT change in ALBI over time attributable to either SBRT (blue) or cirrhosis progression (red), expressed as an absolute change in ALBI score (A) or proportion of the total ALBI change at each timepoint (B). *Abbreviations:* ALBI = albumin-bilirubin score; RT = radiation therapy; SBRT = stereotactic body radiation therapy.

In the mixed model, the mean observed ALBI score after SBRT worsened (increased) from a pretreatment mean of −2.10 (95% CI, −2.18 to −2.01) to −1.88 (95% CI, 1.97 to −1.78) at 6 months. The model predicted that 14.2% of this change was due to cirrhosis alone, whereas 85.8% was due to SBRT (Table 3). At 12 months, the model estimated an overall mean ALBI increase of 0.28; 24.9% was attributed to cirrhosis progression and 75.1% was attributed to SBRT. Model estimates of the absolute and proportional post-SBRT ALBI change attributed to RT versus cirrhosis are shown in Figure 2. In our sensitivity analyses, similar results were obtained after excluding patients with prior liver-directed therapy (n = 105 included in the subset analysis; Table E2), patients who received additional liver-directed therapy after SBRT (n = 68; Table E3), received systemic therapy after SBRT (n = 215; Table E4), and experienced either local or distant intrahepatic tumor progression in the first year after SBRT (n = 124; Table E5). We also performed subset analyses among patients with mean liver minus GTV dose below (Table E6) or above (Table E7) the median physical dose of 7.5 Gy. As expected, the contribution of RT to post-SBRT liver dysfunction was greater for the higher mean liver dose (MLD) group.

## Discussion

In this study, we have used an electronic medical record data set of over 6500 unique patients with cirrhosis to develop a model of the natural history of liver function from the time of cirrhosis diagnosis with the goal of distinguishing the effects of SBRT treatment from the underlying effect of cirrhosis progression. Using this model, we estimated that nearly 25% of the worsening of liver function at 1 year after SBRT is due to cirrhosis. These estimates should improve our ability to model the specific effects of SBRT on liver function and emphasize the need to continue to optimize the medical management of underlying liver dysfunction for these patients.

Deterioration in liver function after SBRT is unfortunately common, with a ≥2-point increase in CP score observed in 10% to 30% of patients within 6 months after SBRT, depending primarily on baseline liver function and liver dose.^13,16^ Before SBRT is delivered, we frequently observe a steady, long-term decline in liver function in patients with HCC consistent with the progression of their underlying cirrhosis.^1,17^ This long-term decline can confound estimates of SBRT toxicity, as posttreatment liver function deterioration could simply represent a continuation of the pretreatment trend. We estimate that, although the majority of posttreatment liver function decline is due to the direct effect of SBRT, a significant proportion (14% and 25% at 6 and 12 months, respectively) is explained by the expected baseline trend. This suggests that previous studies may have overestimated the toxicity of liver SBRT by 10% to 25% depending on the timepoint posttreatment. We performed multiple sensitivity analyses to assess the effect of other clinical factors on our estimates. These sensitivity analyses showed similar results to our primary analysis, suggesting that these factors (other liver-directed therapies, systemic therapy use, and tumor progression/recurrence) were not primary drivers of liver function decline in our cohort.

We chose to measure posttreatment liver function with the ALBI score. The ALBI score was recently developed for assessment of liver function among patients with HCC, for whom liver function is affected by both underlying liver disease and tumor burden, as well as by HCC therapy.^5^ ALBI uses just 2 common laboratory measures, discards arbitrary cutoffs for laboratory values, and eliminates the subjective variables of hepatic encephalopathy and ascites from the CP score. ALBI is becoming more widely accepted as a measure to risk-stratify patients for HCC therapy.^6–8^ Although the prognostic value of ALBI has been well-validated in patients with HCC, population-level trends and trajectories have not been well defined for the larger population of patients with cirrhosis. As 80% to 90% of patients with HCC in many Western countries have underlying cirrhosis, quantifying expected ALBI trends among all patients with cirrhosis is crucial to understanding the incremental impact of HCC and HCC therapies on longitudinal ALBI trajectories. To our knowledge, our study is the first to describe the long-term ALBI trajectories among a large cohort of patients with cirrhosis and will aid the future development of tools for individualized selection of HCC therapy based on future risk of ALBI deterioration.^8^ We observed an improvement of 0.25 points in ALBI score during the year following cirrhosis diagnosis, followed by a slow worsening. This transient improvement may represent the improvement in liver function with initiation of therapies, treatment of underlying liver disease, and improvement in nutrition at the time of cirrhosis diagnosis.

Strengths of our study include the large sample size in the cirrhosis data set, availability of longitudinal laboratory measurements, and a detailed database of SBRT-treated HCC patients. However, there are several limitations. First, we did not have access to cause of death records and therefore could not account for competing (noncirrhosis-related) mortality. Second, our models did not consider worsening in ALBI that could occur from progressive HCC after SBRT. We felt this was a minor factor as 1 year tumor control rates for HCC after SBRT are typically >90%^13^; supporting this assumption, our sensitivity analyses excluding patients with local failures and/or distant intrahepatic failures showed very similar results to our primary analysis. Additional subset analyses excluding patients who underwent systemic therapy and additional liver-directed therapies showed similar results, although we cannot rule out these factors could significantly impact liver function in other cohorts. Third, informative dropout was present in our data, as patients with worse ALBI scores were more likely to have abbreviated follow-up due to their increased risk of death. We used a mixed-effects model with a random effect for each patient to minimize the impact of this issue on individual-level predictions. Fourth, our HCC cohort represents patients from 2 institutions, and diagnosis, treatment, and follow-up patterns in this cohort may not be representative of patients treated at other centers. Fifth, although our sensitivity analyses assessing patient subgroups showed similar results as the primary analysis, sample sizes were limited and a larger validation study could reveal important differences in the relative contribution of radiation to post-SBRT liver dysfunction. Finally, we did not have data on antiviral treatments or alcohol cessation, limiting our ability to comment on the effect of these factors on ALBI trajectory.

In summary, we describe population mean trajectories for ALBI score in cirrhotic patients that permitted us to estimate that ~25% of the overall decrease in liver function that occurs during the first year after SBRT is due to underlying cirrhosis progression. These data can be used to improve efforts for prognostication and modeling of the impact of treatments for HCC on liver function.

## Supplementary Material

1

Supplementary material associated with this article can be found in the online version at doi:10.1016/j.ijrobp.2025.07.1428.

## Figures and Tables

**Table 1:** Characteristics of HCC cohort

Characteristic	N = 260*
Age during RT	64.2 (59.2, 71.3)
Sex	
Female	58 (22.3%)
Male	202 (77.7%)
Race	
African American	24 (9.2%)
Asian	8 (3.1%)
Other	26 (10.0%)
White	200 (76.9%)
Missing	2 (0.8%)
ALBI score (baseline)	−2.15 (−2.56, −1.65)
ALBI grade (baseline)	
1	61 (23.5%)
2	168 (64.6%)
3	31 (11.9%)
Child–Pugh score (baseline)	6 (5–7)
Number of prior liver-directed therapies	
0	105 (40%)
1	58 (22%)
2 or more	97 (38%)
HCC tumor stage	
T1	160 (62%)
T2	65 (25%)
T3	34 (13%)
T4	1 (0.4%)
Maximum tumor dimension (cm)	2.60 (1.80–4.15)
Total number of fractions	3 (3, 5)
Total physical dose (Gy)	36 (33–50)
Mean physical dose to liver-GTV (Gy)	7.5 (4.6–10.3)

*Abbreviations:* ALBI = albumin-bilirubin score; HCC = hepatocellular carcinoma; Gy = gray; RT = radiation therapy; GTV = gross tumor volume.

* Median (IQR); n (%).

**Table 2:** Characteristics of cirrhosis cohort

Characteristic	N = 6789*
Age at cirrhosis diagnosis (y)	64 (56, 71)
Sex	
Female	2914 (43%)
Male	3875 (57%)
Race	
African American	528 (7.8%)
Asian	128 (1.9%)
Other	426 (6.3%)
White	5707 (84%)
Year of cirrhosis diagnosis	
2008–2010	771 (11%)
2011–2013	1749 (26%)
2014–2016	2168 (32%)
2017–2020	2069 (31%)
Cirrhosis type	
Alcoholic	1833 (27%)
HCV	978 (14%)
NOS	3978 (59%)
Liver transplant	529 (7.8%)
HCC diagnosis	466 (6.9%)
Follow-up time (y)	1.70 (0.41,4.14)
Number of ALBI measurements	9 (4, 19)
ALBI (baseline; no units)	−2.19 (−2.79, −1.54)
MELD-Na (baseline; no units)	11 (8, 18)
Albumin (baseline; g/dL)	3.60 (3.00, 4.10)
Total bilirubin (baseline; mg/dL)	1.1 (0.6, 2.5)
AFP (baseline; ng/mL)	4 (3, 7)

*Abbreviations:* AFP = alpha fetoprotein; ALBI = albumin-bilirubin score; HCC = hepatocellular carcinoma; HCV = hepatitis C virus; MELD = model for end-stage liver disease; NOS = not otherwise specified.

* Median (IQR); n (%).

**Table 3:** Post-SBRT ALBI change attributable to radiation toxicity versus cirrhosis progression

Months post-SBRT	Mean observed ALBI (95% CI)	Observed ALBI change (95% CI)	Percent ALBI change due to RT (95% CI)	Percent ALBI change due to cirrhosis (95% CI)
0 (baseline)	−2.095 (−2.177 to −2.013)	-	-	-
3	−1.895 (−1.981 to −1.809)	0.200 (0.140–0.261)	91.2 (88.5–94.0)	8.8 (6.0–11.5)
6	−1.876 (−1.968 to −1.784)	0.251 (0.169–0.333)	85.8 (81.2–90.5)	14.2 (9.5–18.8)
9	−1.857 (−1.958 to −1.755)	0.292 (0.206–0.378)	81.7 (76.2–87.1)	18.3 (12.9–23.8)
12	−1.860 (−1.959 to −1.762)	0.282 (0.169–0.395)	75.1 (65.3–85.0)	24.9 (15.0–34.7)

*Abbreviations:* ALBI = albumin-bilirubin; RT = radiation therapy; SBRT = stereotactic body radiation therapy. ALBI is unitless; higher values represent worse liver function.

## Data Availability

Data is not available for sharing at this time.
