# Supplementary material for: The Contribution of Cirrhosis Progression in Liver Dysfunction After Stereotactic Body Radiation Therapy
Source: Int J Radiat Oncol Biol Phys. Author manuscript; Available in PMC 2026 Jun 12. (PMC13261562; doi:10.1016/j.ijrobp.2025.07.1428)
Supplement: 1 [file NIHMS2182800-supplement-1.docx]

**Supplemental Material for:**

The Contribution of Cirrhosis Progression in Liver Dysfunction After Stereotactic Body Radiation Therapy

**Page 2**: Figure E1. Patient selection diagram for the cirrhosis cohort.

**Page 3**: Figure E2. Cumulative event curves for liver cancer diagnosis and liver transplant.

**Page 4**: Table E1. Regression results of the primary analysis for longitudinal ALBI score.

**Page 5**: Table E2. ALBI change attribution — No prior liver-directed therapies.

**Page 6**: Table E3. ALBI change attribution — No post-SBRT liver-directed therapies.

**Page 7**: Table E4. ALBI change attribution — No systemic therapy.

**Page 8**: Table E5. ALBI change attribution — No tumor progression.

**Page 9**: Table E6. ALBI change attribution — Low mean liver dose (physical dose to Liver-GTV volume less than sample median of 7.5Gy).

**Page 10**: Table E7. ALBI change attribution — High mean liver dose (physical dose to Liver-GTV volume greater than sample median of 7.5Gy).

**Figure E1. Patient selection diagram for the cirrhosis cohort.**

**
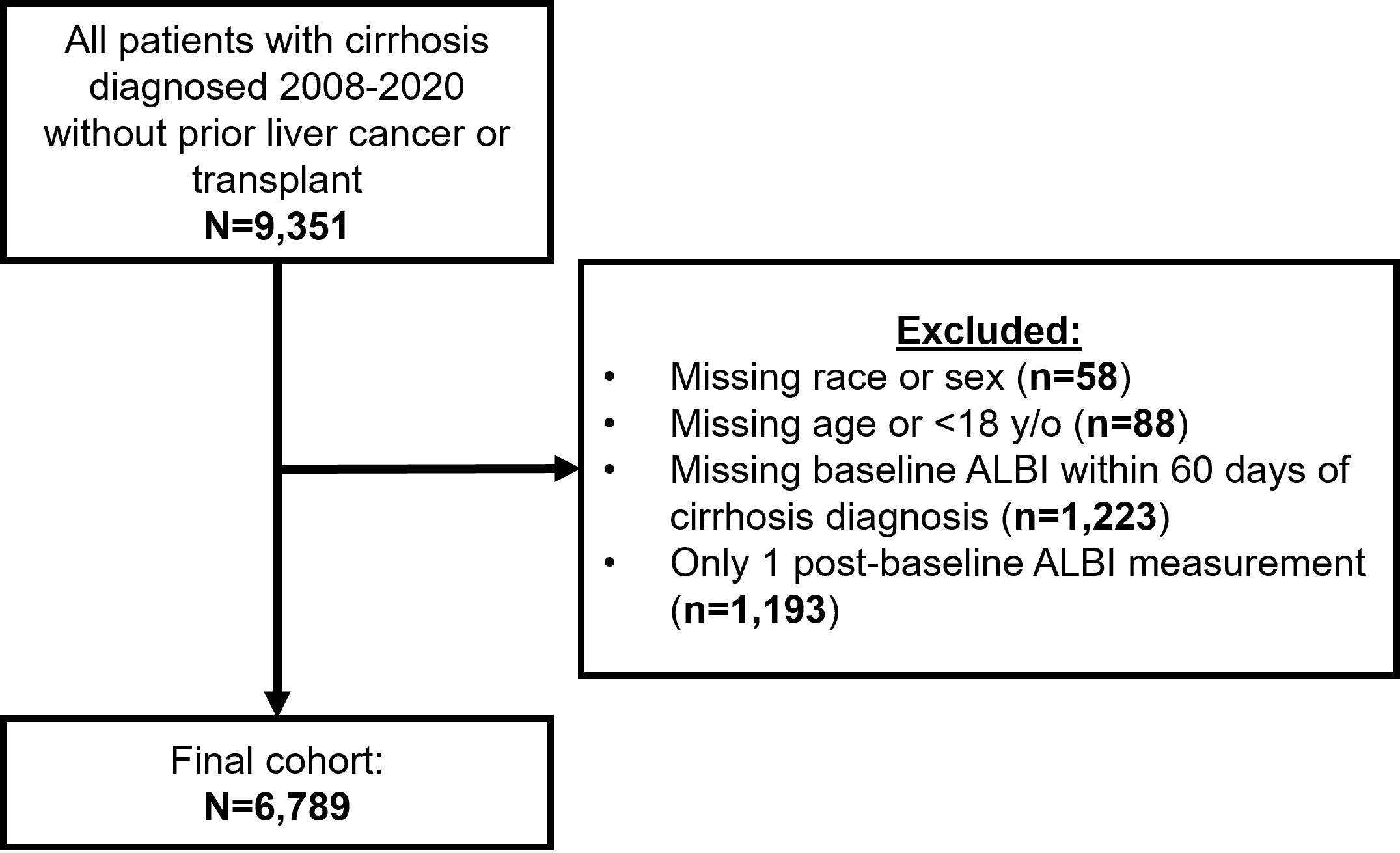
**

**Figure E2.** Cumulative event curves for liver cancer diagnosis and liver transplant in the cirrhosis cohort.

**
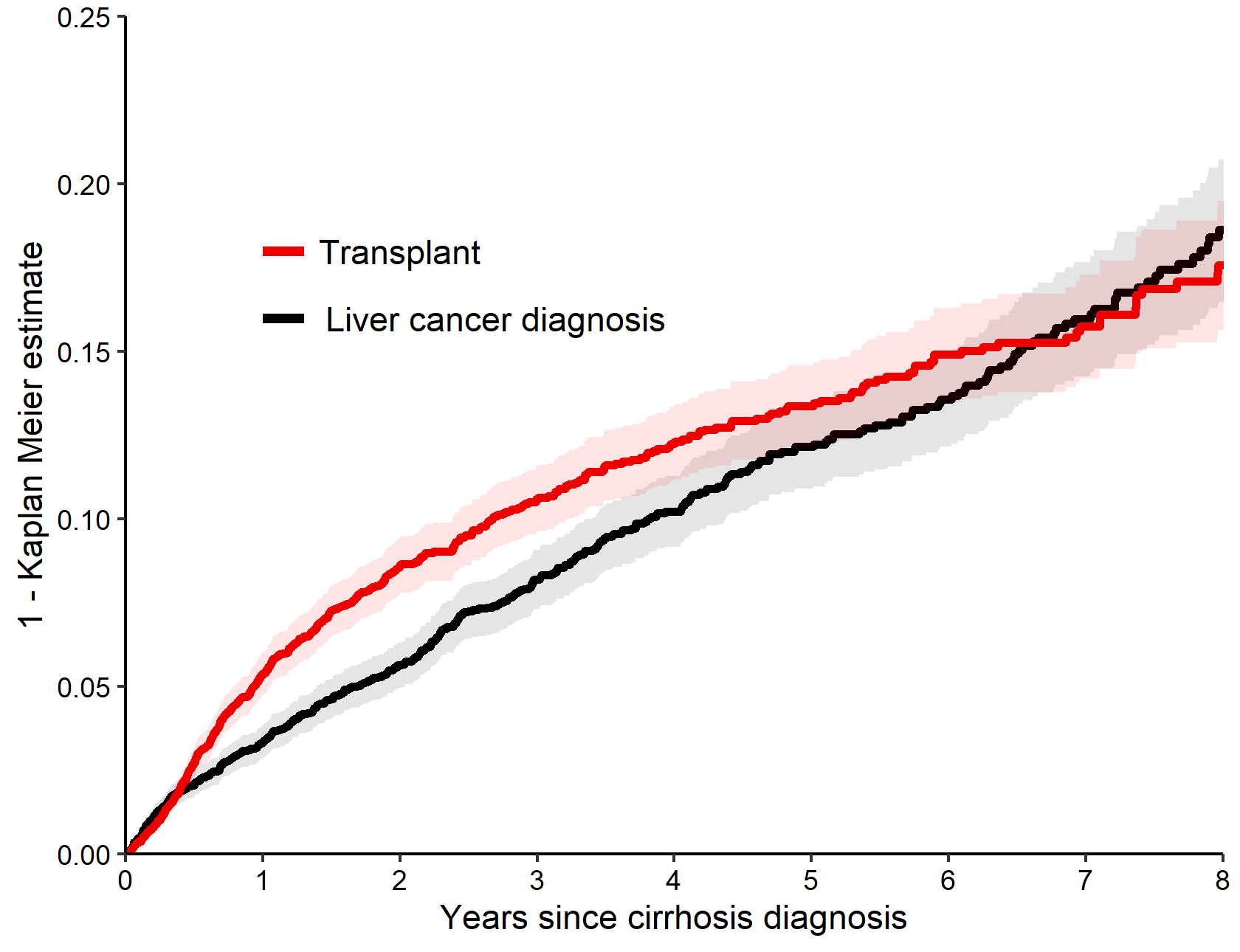
**

Table E1. Regression results of the primary analysis for longitudinal ALBI score

| **Predictors** | **Estimates** | **p-value** |
| --- | --- | --- |
| (Intercept) | -2.003 (-2.032, -1.974) | **<0.001** |
| Male sex | 0.069 (0.032, 0.106) | **<0.001** |
| African American race | -0.046 (-0.115, 0.023) | 0.190 |
| Asian race | -0.438 (-0.578, -0.298) | **<0.001** |
| Other race | 0.120 (0.045, 0.196) | **0.002** |
| Time (linear spline, segment 1) | -0.685 (-0.724, -0.646) | **<0.001** |
| Time (linear spline, segment 2) | 0.073 (0.054, 0.093) | **<0.001** |
| Male sex * Time (linear spline, segment 1) | 0.070 (0.020, 0.120) | **0.006** |
| Male sex * Time (linear spline, segment 2) | 0.004 (-0.020, 0.029) | 0.728 |
| African American race * Time (linear spline, segment 1) | 0.064 (-0.030, 0.157) | 0.183 |
| Asian race * Time (linear spline, segment 1) | -0.073 (-0.273, 0.127) | 0.475 |
| Other race * Time (linear spline, segment 1) | -0.007 (-0.115, 0.101) | 0.894 |
| African American race * Time (linear spline, segment 2) | -0.057 (-0.101, -0.012) | **0.012** |
| Asian race * Time (linear spline, segment 2) | -0.083 (-0.160, -0.005) | **0.036** |
| Other race * Time (linear spline, segment 2) | 0.011 (-0.044, 0.065) | 0.701 |

| **Table E2: ALBI Change Attribution — No Prior Liver-Directed Therapies** | | | | | |
| --- | --- | --- | --- | --- | --- |
|  | **Months post SBRT** | **Mean Observed ALBI (95%% CI)** | **Observed ALBI change (95% CI)** | **Percent ALBI change due to RT (95% CI)** | **Percent ALBI change due to cirrhosis (95% CI)** |
|  | 0 | -2.028 (-2.158, -1.897) | NA (NA, NA) | NA | NA |
|  | 3 | -1.882 (-2.021, -1.742) | 0.146 (0.026, 0.265) | 88.5 (79.0, 98.0) | 11.5 (2.0, 21.0) |
|  | 6 | -1.848 (-1.998, -1.698) | 0.192 (0.048, 0.336) | 82.0 (68.5, 95.4) | 18.0 (4.6, 31.5) |
|  | 9 | -1.840 (-2.004, -1.676) | 0.252 (0.075, 0.428) | 79.1 (64.7, 93.5) | 20.9 (6.5, 35.3) |
|  | 12 | -1.936 (-2.094, -1.777) | 0.184 (-0.011, 0.378) | 62.0 (22.6, 101.4) | 38.0 (-1.4, 77.4) |

| **Table E3: ALBI Change Attribution — No Post-SBRT Liver-Directed Therapies** | | | | | |
| --- | --- | --- | --- | --- | --- |
|  | **Months post SBRT** | **Mean Observed ALBI (95%% CI)** | **Observed ALBI change (95% CI)** | **Percent ALBI change due to RT (95% CI)** | **Percent ALBI change due to cirrhosis (95% CI)** |
|  | 0 | -1.927 (-2.094, -1.759) | NA (NA, NA) | NA | NA |
|  | 3 | -1.802 (-1.985, -1.620) | 0.137 (-0.051, 0.325) | 87.5 (70.6, 104.4) | 12.5 (-4.4, 29.4) |
|  | 6 | -1.806 (-2.012, -1.600) | 0.181 (-0.027, 0.389) | 81.2 (60.2, 102.3) | 18.8 (-2.3, 39.8) |
|  | 9 | -1.805 (-2.078, -1.531) | 0.252 (-0.024, 0.528) | 81.5 (61.9, 101.0) | 18.5 (-1.0, 38.1) |
|  | 12 | -1.947 (-2.198, -1.697) | 0.185 (-0.108, 0.478) | 63.6 (8.5, 118.6) | 36.4 (-18.6, 91.5) |

| **Table E4: ALBI Change Attribution — No Systemic Therapy** | | | | | |
| --- | --- | --- | --- | --- | --- |
|  | **Months post SBRT** | **Mean Observed ALBI (95%% CI)** | **Observed ALBI change (95% CI)** | **Percent ALBI change due to RT (95% CI)** | **Percent ALBI change due to cirrhosis (95% CI)** |
|  | 0 | -2.062 (-2.153, -1.971) | NA (NA, NA) | NA | NA |
|  | 3 | -1.863 (-1.959, -1.768) | 0.201 (0.133, 0.269) | 91.3 (88.2, 94.4) | 8.7 (5.6, 11.8) |
|  | 6 | -1.839 (-1.942, -1.737) | 0.254 (0.172, 0.337) | 86.0 (81.5, 90.6) | 14.0 (9.4, 18.5) |
|  | 9 | -1.839 (-1.951, -1.726) | 0.285 (0.196, 0.373) | 81.5 (75.8, 87.2) | 18.5 (12.8, 24.2) |
|  | 12 | -1.859 (-1.969, -1.749) | 0.276 (0.143, 0.410) | 75.0 (63.1, 86.9) | 25.0 (13.1, 36.9) |

| **Table E5: ALBI Change Attribution — No Tumor Progression** | | | | | |
| --- | --- | --- | --- | --- | --- |
|  | **Months post SBRT** | **Mean Observed ALBI (95%% CI)** | **Observed ALBI change (95% CI)** | **Percent ALBI change due to RT (95% CI)** | **Percent ALBI change due to cirrhosis (95% CI)** |
|  | 0 | -2.048 (-2.172, -1.924) | NA (NA, NA) | NA | NA |
|  | 3 | -1.879 (-2.009, -1.749) | 0.167 (0.083, 0.251) | 89.5 (84.1, 94.8) | 10.5 (5.2, 15.9) |
|  | 6 | -1.865 (-2.004, -1.727) | 0.213 (0.102, 0.325) | 83.3 (74.6, 91.9) | 16.7 (8.1, 25.4) |
|  | 9 | -1.872 (-2.026, -1.718) | 0.250 (0.119, 0.381) | 78.9 (68.1, 89.8) | 21.1 (10.2, 31.9) |
|  | 12 | -1.930 (-2.078, -1.782) | 0.159 (-0.020, 0.338) | 55.6 (7.2, 104.0) | 44.4 (-4.0, 92.8) |

| **Table E6: ALBI Change Attribution — Low Mean Liver Dose (physical dose to Liver-GTV volume less than sample median of 7.5Gy).** | | | | | |
| --- | --- | --- | --- | --- | --- |
|  | **Months post SBRT** | **Mean Observed ALBI (95%% CI)** | **Observed ALBI change (95% CI)** | **Percent ALBI change due to RT (95% CI)** | **Percent ALBI change due to cirrhosis (95% CI)** |
|  | 0 | -1.995 (-2.113, -1.876) | NA (NA, NA) | NA | NA |
|  | 3 | -1.858 (-1.980, -1.735) | 0.148 (0.077, 0.220) | 87.9 (82.0, 93.8) | 12.1 (6.2, 18.0) |
|  | 6 | -1.798 (-1.927, -1.670) | 0.215 (0.118, 0.312) | 82.9 (75.2, 90.6) | 17.1 (9.4, 24.8) |
|  | 9 | -1.811 (-1.948, -1.674) | 0.241 (0.135, 0.347) | 77.4 (67.7, 87.2) | 22.6 (12.8, 32.3) |
|  | 12 | -1.832 (-1.964, -1.701) | 0.216 (0.078, 0.355) | 66.9 (46.1, 87.7) | 33.1 (12.3, 53.9) |

| **Table E7: ALBI Change Attribution — High Mean Liver Dose (physical dose to Liver-GTV volume greater than sample median of 7.5Gy).** | | | | | |
| --- | --- | --- | --- | --- | --- |
|  | **Months post SBRT** | **Mean Observed ALBI (95%% CI)** | **Observed ALBI change (95% CI)** | **Percent ALBI change due to RT (95% CI)** | **Percent ALBI change due to cirrhosis (95% CI)** |
|  | 0 | -2.188 (-2.305, -2.071) | NA (NA, NA) | NA | NA |
|  | 3 | -1.929 (-2.055, -1.803) | 0.251 (0.152, 0.351) | 93.2 (90.2, 96.1) | 6.8 (3.9, 9.8) |
|  | 6 | -1.958 (-2.095, -1.822) | 0.284 (0.145, 0.423) | 88.0 (82.1, 93.9) | 12.0 (6.1, 17.9) |
|  | 9 | -1.902 (-2.060, -1.744) | 0.324 (0.172, 0.476) | 83.9 (76.4, 91.4) | 16.1 (8.6, 23.6) |
|  | 12 | -1.885 (-2.042, -1.727) | 0.332 (0.137, 0.526) | 79.6 (68.0, 91.2) | 20.4 (8.8, 32.0) |
